#  A role of the 53BP1 protein in genome protection: structural and functional characteristics of 53BP1-dependent DNA repair

**DOI:** 10.18632/aging.101917

**Published:** 2019-04-17

**Authors:** Eva Bártová, Soňa Legartová, Miroslav Dundr, Jana Suchánková

**Affiliations:** 1 Institute of Biophysics of the Czech Academy of Sciences, 612 65 Brno, Czech Republic; 2 Rosalind Franklin University of Medicine and Science, Chicago Medical School, Department of Cell Biology, North Chicago, IL 60064, USA

**Keywords:** 53BP1, BRCA1, DNA damage, histone modifications, epigenetics

## Abstract

Nuclear architecture plays a significant role in DNA repair mechanisms. It is evident that proteins involved in DNA repair are compartmentalized in not only spontaneously occurring DNA lesions or ionizing radiation-induced foci (IRIF), but a specific clustering of these proteins can also be observed within the whole cell nucleus. For example, 53BP1-positive and BRCA1-positive DNA repair foci decorate chromocenters and can appear close to nuclear speckles. Both 53BP1 and BRCA1 are well-described factors that play an essential role in double-strand break (DSB) repair. These proteins are members of two protein complexes: 53BP1-RIF1-PTIP and BRCA1-CtIP, which make a “decision” determining whether canonical nonhomologous end joining (NHEJ) or homology-directed repair (HDR) is activated. It is generally accepted that 53BP1 mediates the NHEJ mechanism, while HDR is activated via a BRCA1-dependent signaling pathway. Interestingly, the 53BP1 protein appears relatively quickly at DSB sites, while BRCA1 is functional at later stages of DNA repair, as soon as the Mre11-Rad50-Nbs1 complex is recruited to the DNA lesions. A function of the 53BP1 protein is also linked to a specific histone signature, including phosphorylation of histone H2AX (γH2AX) or methylation of histone H4 at the lysine 20 position (H4K20me); therefore, we also discuss an epigenetic landscape of 53BP1-positive DNA lesions.

## Introduction

**Figure 1:**
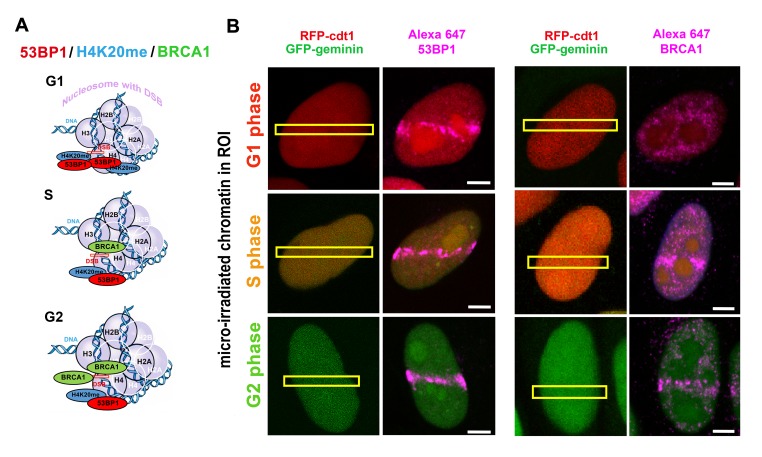
(**A**) Illustration of the levels of 53BP1, H4K20me2/me3 and BRCA1 in the G1, S and G2 phases of the cell cycle. (**B**) An example of recruitment of the 53BP1 protein and BRCA1 (both violet) to locally induced DNA lesions by laser microirradiation in the G1 (red), S (orange) and G2 cell cycle phases. HeLa-Fucci cells that expressed RFP-tagged Cdt1 (red) in the G1 phase and GFP-tagged geminin in the G2 phase were used. This figure shows maximal projection from 60 optical sections (in each example) and provides a pictorial illustration of the 53BP1 and BRCA1 levels in the G1, S, and G2 phases of the cell cycle as published elsewhere. Due to both RFP-ctd1 and GFP-geminin expression, cells were fixed by formaldehyde, and immunostaining with an antibody against 53BP1 or BRCA1 was performed (see Alexa-647 stained 53BP1 or BRCA1). For methodology explaining immunofluorescence and the use of local laser microirradiation, see [161].

Cells have evolved multiple conserved mechanisms for maintaining genome integrity, which is collectively termed the DNA damage response (DDR). These mechanisms enable cells to identify and repair different types of DNA lesions, including deleterious double-strand breaks (DSBs). It is evident that error-prone repair of DSBs can lead to changes in the genome, including chromosomal translocations or complex chromosome rearrangements. Subsequently, on the cellular level, a disorder in the genome induces uncontrolled cell proliferation, which is the main characteristic of malignant cells. The following very conservative and mechanistically distinct repair pathways are activated in the cell nucleus: the quick but error-prone nonhomologous end joining (NHEJ) and the more accurate homology-directed repair (HDR). It is generally accepted that NHEJ repair is the main pathway recognizing DSB sites in the G1 phase of the cell cycle. Both NHEJ and HDR mechanisms recognize DSBs in S, G2, and M phases of the cell cycle; however, HDR has a dominant role in this repair process [1]. It is well known that a “choice” between NHEJ and HDR is mediated via 53BP1 and BRCA1 proteins that accumulate asymmetrically at DNA lesions in different phases of the cell cycle. NHEJ-related repair factor 53BP1 binds to H4K20me2/me3, which is abundant at DSBs appearing in the G1 phase of the cell cycle. On the other hand, the BRCA1 level is elevated at DSB sites of the cells in the S and G2 phases ([2–4]; see illustration in Figure 1A, B). A critical stage for BRCA1-directed HDR is the S phase when the DNA replication process generates a sister chromatid that acts as a template for the complementary DNA strand. However, Kakarougkas and Jeggo [5] have suggested that when DNA repair processes are activated in S/G2 phases, the mechanism of the first response is not, surprisingly, HDR, but rather NHEJ repair. On the cell population level, the preference for NHEJ signaling may also be expected because terminally differentiated cells are arrested in G0/G1, the phases when NHEJ repair plays a dominant role. Additionally, in *in vitro* cultivated cell populations, the highest number of cells occur in the G1 phase; thus, NHEJ repair should preferentially be initiated in the majority of these cycling cells. On the other hand, many irradiated cell populations are characterized by cell cycle rearrangement and arrest in the G2 phase [6–8]. Therefore, DSBs in cells exposed to ionizing radiation could be preferentially repaired via BRCA1-mediated HDR.

**Figure 2:**
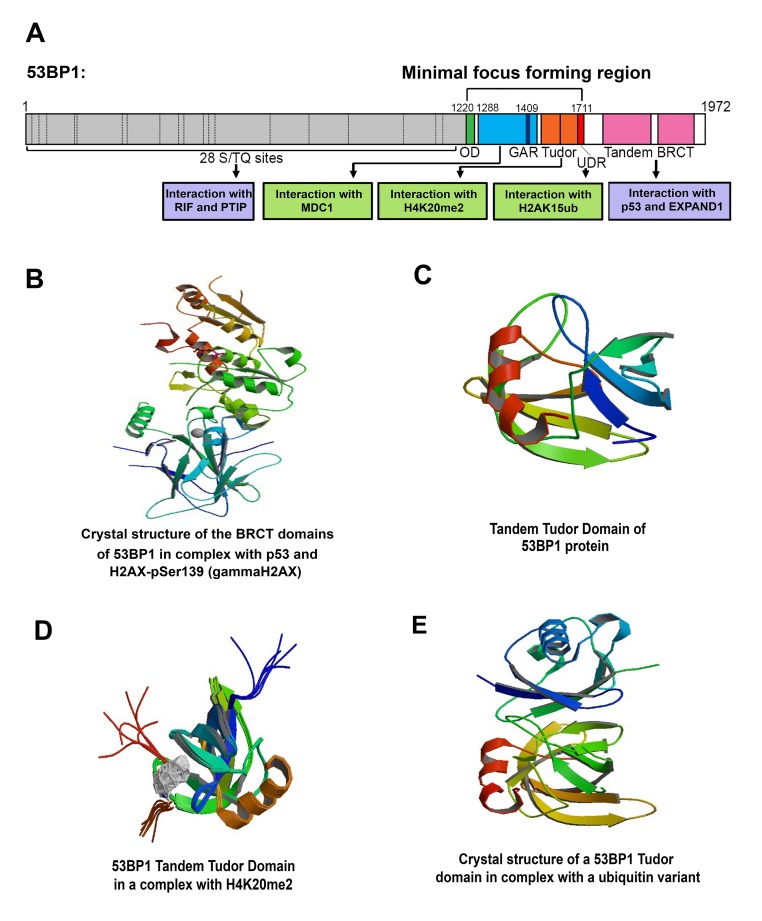
(**A**) Structural domains of the 53BP1 protein and its interaction partners (partially adapted from [101]). (**B**) Crystal structure of the BRCT domains of 53BP1 in complex with p53 and H2AX-pSer139 (γH2AX); (adapted from PDB protein database, authors: Day M., Oliver, A.W., Pearl, L.H. (**C**) Structure of tandem Tudor domains (http://www.rcsb.org/3d-view/1XNI/1 [107];). (**D**) A tandem Tudor domain of the 53BP1 protein in complex with H4K20me2 (http://www.rcsb.org/structure/2LVM [100];). (**E**) Crystal structure of a 53BP1 Tudor domain in complex with a ubiquitin variant (http://www.rcsb.org/structure/5J26; author: Wan et al., to be published). The structural data in panels B-E were derived from the Protein Data Bank (PDB).

**Figure 3:**
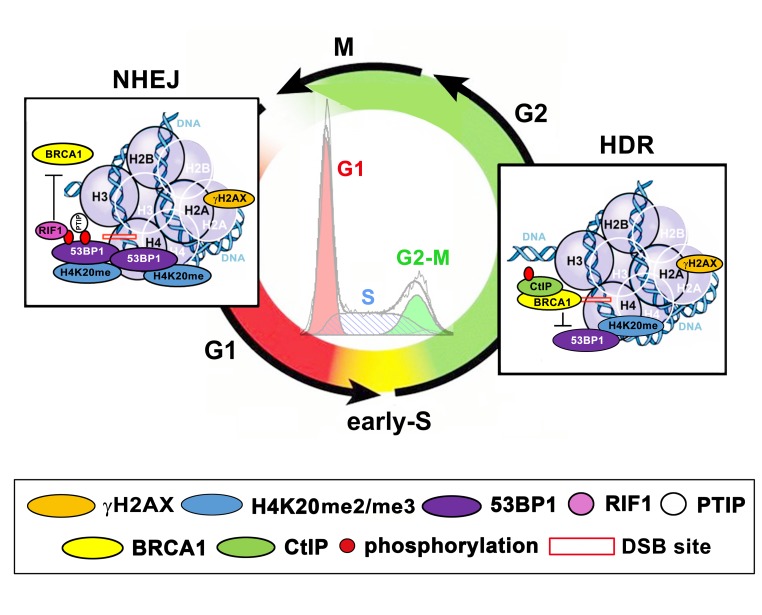
A schematic illustration of the functions of the H4K20me2/me3-53BP1-RIF1-PTIP and BRCA1-CtIP protein complexes in the G1 and G2 phases of the cell cycle. DSB sites are depicted by red frames. The G1 phase is shown in red, the S phase is shown in yellow-orange (dashed blue in histogram from flow cytometry), and the G2 phase is shown in green (see the circular graph).

As mentioned above, it is well known that the 53BP1 protein and its specific domains (Figure 2A-E) are considered to be essential factors for the NHEJ repair mechanism. Moreover, 53BP1 together with telomere homeostasis maintenance factor, RIF1, is believed to form a barrier inhibiting DNA end resection. Zimmermann et al. [9] and Fontana et al. [10] summarized that RIF1 inhibits 5’ end resection and activation of HDR factors such as CtIP and DNA helicase BLM. Also, RIF1 function prevents the recruitment of BRCA1/BARD1 proteins to damaged chromatin. These data unambiguously show that the tumor suppressor BRCA1 acts in an opposing way on 53BP1-RIF1 proteins [3]. Identically, Escribano-Diaz et al. [11] specified the DNA repair pathway choice regulated by the 53BP-RIF1 and BRCA1-CtIP protein complexes. These authors suggest that BRCA1 activity is mostly directed to the regulation of 53BP1 function in the S/G2 phases of the cell cycle. Moreover, 53BP1 together with RIF1 have been shown to prevent BRCA1 accumulation at DSBs that appear in the G1 phase of the cell cycle (refer to Figure 3). In general, it is evident that not only 53BP1 and BRCA1 but also 53BP1-RIF1 and BRCA1-CtIP protein complexes represent important factors that make a “decision” regarding whether the NHEJ or HDR pathways will be initiated (Figure 3). Data on the dominant function of BRCA1 in the competition between NHEJ and HDR are not consistent [12–14]. However, a consensus is that BRCA1 is considered as a scaffold protein that enables the recruitment of other proteins to DSB sites [15]. Surprisingly, the BRCA1 protein itself is recruited to damaged chromatin relatively late, 30 minutes after genome injury [16,17]. The initiation step of HDR is primarily ascribed to the MRN complex and/or the CtIP protein. Then, replication protein A (RPA) binds to 3’ single-stranded DNA (ssDNA) that is generated by nucleolytic degradation of the 5’ strands. Subsequently, via the function of BRCA2, the RPA protein is replaced by Rad51, and thus Rad51-ssDNA nucleoprotein filaments are created. This is the first step of HDR that leads to physiological DNA repair, in which BRCA1 is presumably engaged in the later stages due to delay in the recruitment kinetics. On the other hand, it is generally accepted that BRCA1 promotes DNA end resection by recruiting the CtIP protein to DSBs [18–21].

An integral part of DNA repair mechanisms also includes specific histone posttranslational modifications (PTMs), such as γH2AX, H4K20 di-/trimethylation (H4K20me2/me3) and H2AK15 ubiquitination (H2AK15ub) (summarized by [4,22,23]). In the majority of cases, histone markers serve as an epigenetic scaffold that is recognized by specific DNA repair proteins [24]. A significant interaction has been identified between H4K20me2 and the 53BP1 protein; however, an interaction between γH2AX and 53BP1 is not widely accepted. On the other hand, Kleiner et al. [25] showed that γH2AX binders, along with the 53BP1 protein, recognize phosphorylation of H2AX via its BRCT domain (Figure 2B). One such binder, Ataxia telangiectasia mutated (ATM) kinase, is not only responsible for γH2AX but also for the phosphorylation of the 53BP1 protein. This epigenetic process is essential for canonical NHEJ repair [26]. Moreover, Gupta et al. [27] recently described the ATM-RNF8-RNF168-53BP1 cascade that promotes the NHEJ-related repair of intrachromosomal breaks. Feng et al. [3] summarized that ATM-dependent 53BP1 phosphorylation plays a role exclusively in the G1 phase of the cell cycle. In this case, RIF1 and PTIP recognize the phosphorylated form of the 53BP1 protein [28] (Figure 3). Isono et al. [29] further indicated that the 53BP1-dependent protein complex is interrupted when BRCA1 promotes DNA end resection. These authors reported that the phosphatase PP4C has a significant role in 53BP1 dephosphorylation and RIF1 release from the protein complex that recognizes chromatin with DSBs. The process by which BRCA1 promotes PP4C-dependent 53BP1 dephosphorylation is considered a crucial step of HDR that is accompanied by a BRCA1 interaction with phosphorylated CtIP at serine residue S327 [30]. This phosphorylation event is mediated via cyclin-dependent kinase 1 (CDK 1) [31,32]. However, recent evidence indicates that the interaction between BRCA1 and CtIP in a phospho-dependent manner is not an essential step for HDR-mediated DSB repair [33]. Polato et al. [34] showed an independent function between CtIP and BRCA1 in promoting DSB end resection. Interestingly, the loss of CtIP-BRCA1 interaction does not disturb genome stability.

The efficiency of DNA repair is also affected by acetylation of the 53BP1 protein. This posttranslational modification inhibits NHEJ and activates HDR via the negative regulation of 53BP1 accumulation in chromatin with DSBs [35]. In brief, the acetylated 53BP1 protein loses its ability to bind to damaged nucleosomes and thus, together with PTIP and RIF1, does not move to DSB sites. This process is mediated by histone deacetylase 2 (HDAC 2), whose function seems to also be important in the “choice” between the NHEJ and HDR mechanisms. Together, these observations show that not only the posttranslational modifications of histones but also the PTMs of DNA repair proteins, including 53BP1, are essential for the regulation of DNA repair processes.

Interestingly, DNA repair proteins are also functional during mitotic cell division, though to a reduced extent. Nevertheless, DNA repair processes are significantly downregulated in this cell cycle stage. Literature sources show that mechanisms responsible for DSB repair in mitosis are limited. For example, Peterson et al. [31] showed that DNA-end resection in the M-phase is associated with Mre11-Rad50-Nbs1(MRN)-CtIP activation, and this process is not associated with ATR- or Rad51 function. In this DNA repair pathway, CDK1 is responsible for the phosphorylation of CtIP, which likely prevents the binding of Rad51 to the DNA strand [31]. In the M phase of the cell cycle, the DNA repair proteins are characterized by a specifically localized morphology. For example, the BRCA1 protein colocalizes with the centrosome [36], and the 53BP1 protein does not accumulate in DNA-damaged foci. Instead, mitotic DNA repair foci are positive for the MDC1 protein, and the MRN protein complex and/or these foci are characterized by phosphorylation of histone H2AX [37,38]. Interestingly, mitotic kinases phosphorylate the 53BP1 protein and RNF8 (the E3 ubiquitin ligase), but neither of those DNA repair factors is recruited to DSB sites on mitotic chromosomes. When the focal accumulation of these DNA repair proteins is experimentally restored, the mitotic DNA repair machinery instead initiates undesirable fusion of sister telomeres, which leads to the formation of dicentric chromosomes and aneuploid cells [39].

Lukas et al. [40] additionally showed that replication stress increases the number of DNA repair foci that are well visible during mitosis and subsequently in both daughter cells in the G1 phase of the cell cycle. These authors observed that the number of 53BP1-positive foci increased after depletion of a DNA helicase BLM. However, the number of these foci was reduced when the SMC2 protein, a member of the condensin complex, was depleted [40].

### Comparison of 53BP1 protein functions in the regulation of transcription and DNA repair: a functional link between 53BP1 and the p53 protein

**Figure 4:**
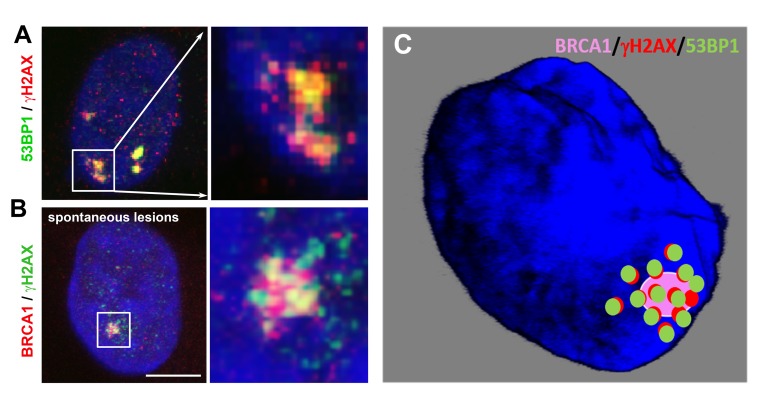
(**A**) Nuclear arrangement of the 53BP1 (green) protein and γH2AX (red) in spontaneously occurring DNA lesions. (**B**) The nuclear distribution pattern of the BRCA1 protein (red) and γH2AX (green) in spontaneous DNA lesions. HeLa cells were used for these illustrations, which depict the primary results published by [67] and [20]. Scale bars, 10 µm. **(C**) A pictorial illustration of BRCA1/γH2AX/53BP1 compartmentalization at a spontaneous DSB site: Chapman et al. [67] showed a spatial link between 53BP1- and BRCA1-positive foci or 53BP1- and γH2AX-positive repair foci. Suchánková et al. [20] described the methodology of immunostaining and showed a degree of colocalization between 53BP1-γH2AX, 53BP1-MDC1, and MDC1-γH2AX.

It is well known that the 53BP1 protein (Figure 2A) binds to the p53 protein in order to regulate cell cycle progression and cell proliferation [41]. It is believed that the p53 protein has a transcription-dependent and independent function in both nucleotide excision repair (NER) and base excision repair (BER) [42]. However, p53 in itself does not recruit to DNA lesions (our unpublished observation), but instead regulates DNA repair process indirectly, via halting the cell cycle and/or inducing mitochondrial pathway of apoptosis that is characterized by oligonucleosomal fragmentation. On the other hand, the 53BP1 protein plays a direct role in NHEJ-dependent repair of DSB sites. Immunofluorescence analysis indicated the existence of three pools of 53BP1: (1) a cytoplasmic protein fraction, (2) a homogeneously dispersed nuclear fraction, and (3) body-like nuclear structures, referred to as DNA repair foci [43]. The induction of DSBs induces changes in the 53BP1 protein nuclear distribution, resulting in the reorganization of fractions (2) and (3). Nuclear rearrangement, induced by radiation or DNA-damaging agents, involves a shift from a diffuse nuclear localization of 53BP1 to discrete foci, which, for example, colocalize with phosphorylated histone H2AX [44–46] (Figure 4A).

53BP1 belongs to a family of evolutionarily conserved DNA damage checkpoint proteins and is the vertebrate ortholog of the budding yeast Rad9 and fission yeast Crb2/Rhp9 checkpoint proteins. 53BP1 is a protein that consists of 1972 amino acids and lacks enzymatic activities directly implicated in DNA repair. This protein interacts with numerous other factors that recognize DSBs or related histone signatures. 53BP1 has several important structural domains, including BRCT repeats located at the C-terminus, tandem Tudor domain (TTD), and 28 amino-terminal Ser/Thr-Gln phosphorylation sites, which are phosphorylated by the ATM kinase (Figure 2A, C). ATM-mediated phosphorylation of the N-terminus of 53BP1 recruits its well-known downstream effectors, including PTIP and RIF1. The C-terminus of 53BP1 contains two BRCT (BRCA1 C-terminus) domains that bind to the DNA-binding domain of tumor suppressor p53. Moreover, 53BP1 likely binds to γH2AX or a chromatin-bound factor EXPAND1 [25] (Figure 2A, B). However, the concept of direct interaction between 53BP1 and γH2AX is not widely accepted. Some experiments have shown that a function of the 53BP1 protein is linked to γH2AX [46,47], but other authors did not confirm a functional link between 53BP1 and γH2AX [48,49]. It is believed that the binding of 53BP1 to DSB sites depends on its ability to recognize either H4K20me2 or H2AK13/K15ub. These epigenetic marks appear downstream from γH2AX-positive DNA lesions. Baldock et al. [50] showed that in contrast to the current H4K20me2-dependent or H2AK13/K15ub-dependent models, the third possibility of how the 53BP1 protein functions at DNA lesions is its binding to γH2AX via the BRCT domain (Figure 2B). Moreover, a very important region of the 53BP1 protein is the Minimal focus-forming region that is responsible for the spatial arrangement of DNA repair foci. This region contains an N-terminal oligomerization domain (OD), MDC1-binding region, glycine-arginine rich (GAR) motif, tandem Tudor motif, and ubiquitylation-dependent recruitment (UDR) motif that interacts with histone H2AK15ub (Figure 2A, C-E). This central region is required not only for the formation of 53BP1-positive repair foci but also for 53BP1 binding to the kinetochore.

Iwabuchi et al. [41] further showed that 53BP1 has the ability to recognize wild-type but not mutant p53 protein. This selection process is mediated by the DNA-binding domain of p53 and is primarily linked to gene-silencing processes [43]. Recently, we investigated the function of both 53BP1 and p53 proteins in DNA damage repair. We analyzed whether mutations in the TP53 gene (encoding the p53 protein) can change the recruitment kinetics of the 53BP1 protein to locally microirradiated chromatin [20]. We also investigated whether distinct mutations in the TP53 gene affect the interaction between p53 and 53BP1 proteins. In this study, we observed that the TP53 hot spot mutation in the DNA binding surface (R273C) weakens an interaction between p53 and 53BP1, whereas a TP53 mutation at the DNA-binding site (L194F) does not affect the mutual interaction between p53 and 53BP1. Interestingly, the deletion of the TP53 gene completely abrogated the interaction between the 53BP1 and MDC1 proteins, and distinct mutations in the TP53 gene were associated with different recruitment kinetics of the 53BP1 protein to locally microirradiated chromatin [20]. These results implied a direct link between the DDR-related function of 53BP1 and cytogenetic changes in the TP53 gene. However, an exact 53BP1-p53-dependent DNA repair mechanism remains elusive [51]. We showed that a cancer hot spot mutation in the DNA binding surface (R282W) leads to the early recruitment of 53BP1 to locally induced DNA lesions; however, TP53-mutant cells (L194F) experienced a 60-70 minute delay in the appearance of 53BP1 protein at the microirradiated region of the cell nucleus. This delayed recruitment of the 53BP1 protein at UVA-damaged chromatin is not typical for cells with a normal diploid karyotype, including human IMR90 fibroblasts and mouse embryonic fibroblasts (MEFs), which are characterized by an immediate appearance of the 53BP1 protein at DSB sites [20]. In contrast, human aneuploid cervical carcinoma (HeLa) cells are characterized by a late accumulation of the 53BP1 protein at UVA-damaged chromatin. In these cells, the 53BP1 protein is recruited to irradiated chromatin approximately 10 minutes after cell exposure to a radiation source, and 53BP1 remains at the irradiated chromatin for up to 90 minutes [20]. In contrast, BRCA1 is recruited to the lesions 25-30 minutes after local laser microirradiation, which represents a very late DNA damage response [16,17]. The described localized kinetics of 53BP1 and BRCA1 proteins were observed in UV-induced DNA lesions that were mainly positive for cyclobutane pyrimidine dimers (CPDs). However, DSBs appear at UV-damaged chromatin, especially in the case of high-dose laser exposure. It is generally accepted that local laser microirradiation induces a mixture of various lesions in the genome, but the same is also true for γ-irradiation [52]. For these reasons, we recently optimized local laser microirradiation to avoid the appearance of CPDs. For studies of DSB repair by the microirradiation procedure, we recommend the use of a 405-nm laser line working near UV light. Local microirradiation must be performed in the absence of BrdU (bromodeoxyuridine) or Hoechst 33342 presensitization [52].

Together, literature sources showed that protein diffusion kinetics might be affected by the type and intensity of radiation source. This fact must be considered. The observations discussed here reflect distinct DDR-related functions of 53BP1 and BRCA1 proteins and indicate that genome instability, including TP53 mutations, may affect the localized kinetics of DDR-related proteins. It is evident that the co-regulatory function of 53BP1 and p53, primarily crucial for transcription, could also play a role in the DNA damage response because mutations in the TP53 gene may affect the recruitment kinetics of 53BP1 to UVA-damaged chromatin [20]. This observation confirms that both p53 and 53BP1 represent cellular guardians of physiological nuclear processes, and DNA repair is not an exception. Thus, disorders in these proteins lead to pathophysiological states.

### Nuclear arrangement and localized kinetics of 53BP1-positive DNA repair foci

**Figure 5:**
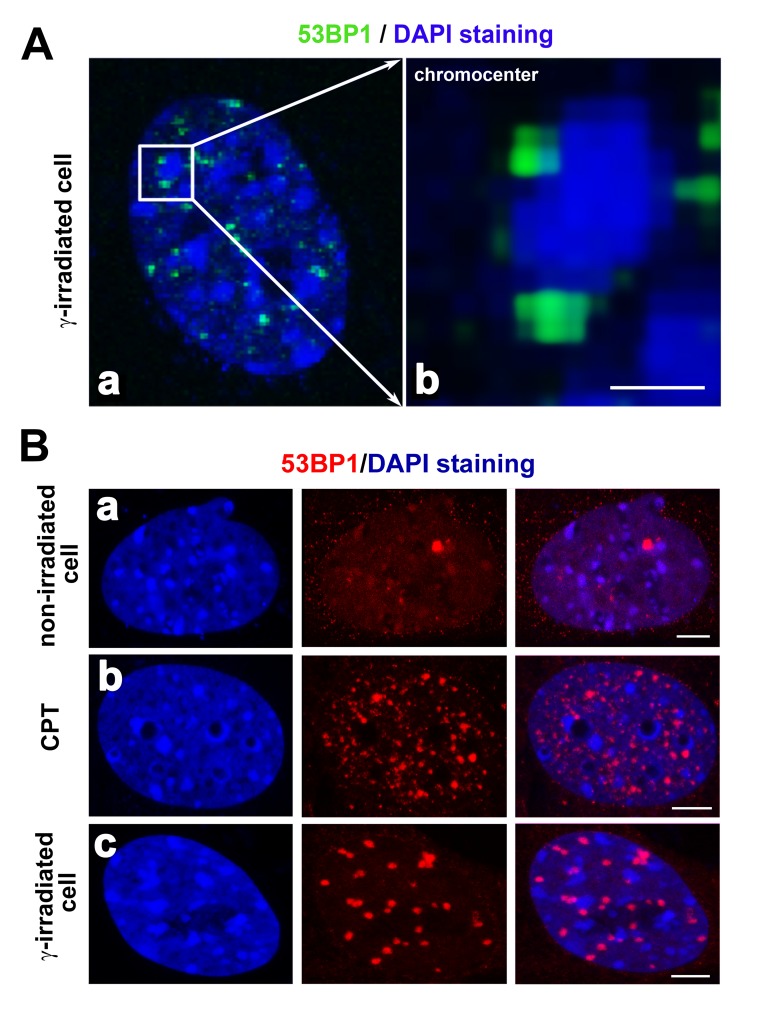
(**A**) Localization of the 53BP1 protein (green) in close proximity to chromocenters (clusters of centromeric heterochromatin; blue) is shown. Reindl et al. [68] showed that the 53BP1 protein localized in close proximity to the perichromatin region. This picture is our illustration of 53BP1 localization at the periphery of chromocenters. Here, DAPI was used for the visualization of MEF nuclei. In panel (**A**), chromocenters are characterized by dense DAPI staining. Panel (**a**) shows the DAPI-stained interphase nucleus and (**b**) is the magnified chromocenter (blue) decorated by 53BP1-positive foci (green). The scale bar represents 1 µm. (**B**) Compared to (**a**) non-irradiated cells, (**b**) tiny DNA damage foci may be induced by camptothecin (CPT) treatment. The 53BP1 protein (red) did not overlap with chromocenters in **(c)** γ-irradiated cells. The 53BP1 foci of CPT-treated cells were characterized by a distinct morphology compared to IRIF. A number of foci may be different in distinct cell lines and after cell exposure to distinct types of radiation or DNA damaging agents, as shown by [80] or [53], and see here. Scale bars in panels Ba-c represent 5 µm.

The recruitment of 53BP1 to spontaneous repair foci, ionizing radiation-induced foci (IRIF) or DNA lesions induced by DNA damaging agents is a characteristic structural feature of NHEJ repair machinery (Figure 4A-C, Figure 5A, 5Ba-c). The kinetics of fluorescently tagged proteins, accumulated at DNA repair foci can be studied, for example, by time-lapse confocal microscopy combined with single-particle tracking analysis, and another very useful tool for these studies is the fluorescence recovery after photobleaching (FRAP) technique. With regard to protein diffusion kinetics, we have shown that the mCherry-tagged 53BP1 protein recovers more rapidly in UV-induced DNA lesions than in spontaneous DNA repair foci [53]. For other DNA repair proteins, Hable et al. [54] revealed that RAD52 mobility is slower than MDC1 mobility. Notably, MDC1 recruitment to DNA lesions after UV laser irradiation resembles the recruitment of MDC1 to DSB sites induced by high LET‑ionizing radiation rather than low LET-ionizing radiation. This work demonstrates that the extent of DNA damage and a type of radiation source have a significant influence on repair processes and should be considered when comparing different experimental studies. Additionally, the magnitude and type of DNA injury must be taken into consideration when evaluating the localized kinetics of DNA repair proteins in living cells. Moreover, the localized kinetics of exogenous protein in DNA lesions must be verified at the endogenous protein level [55,56].

An example of protein mobility showed by Lottersberger et al. [57] documented the 53BP1/LINC/microtubule-dependent mobility of IRIF. These authors suggested two model mechanisms that regulate the local motion of DNA repair foci. The first model involves a mechanism in which 53BP1 interacts with the linker of the nucleoskeleton and cytoskeleton complex (LINC) [58]. In this case, the kinesin- and microtubule-dependent mobility of LINC affects the localized dynamics of DSB sites. In the second model, no interaction between 53BP1 and LINC is considered, and the LINC complex transduces microtubule forces in an untargeted manner. Similarly, Mekhail [59] summarized that the disruption of motor proteins or robust microtubules leads to disorder in the localized movement of damaged DNA.

From the view of cellular structures, we recently identified a radiation-induced constrained local motion of 53BP1-positive foci that colocalized with promyelocytic leukemia (PML) nuclear bodies. The movement trajectory of PML-53BP1-positive DNA repair foci was reduced compared to those of individual PML bodies [60]. We also identified reduced local motion for Cajal bodies (CBs) in γ-irradiated cells when these cells were compared with their nonirradiated counterparts [61]. In general, Becker et al. [62] suggested that DSB-positive foci are characterized by limited local motion on a limited spatial scale. This constrained motion is dependent on a functional ATM kinase, and the localized kinetics of radiation-induced foci were subtly affected by the depletion of chromatin remodeling- and DNA binding proteins. Interestingly, the dynamic properties of DSB sites leading to chromosomal translocation have been reported by [63]. These authors showed a cell cycle-independent occurrence of chromosome translocations that appeared over hours as a consequence of incorrectly repaired DSB sites.

Together, FRAP, time-lapse microscopy, and single-particle tracking analyses represent a valuable biophysical approach for studying the dynamic properties of DNA repair proteins [60,63–66]. These experimental tools, which are useful for live cell studies, are essential for understanding the functional properties and hierarchical binding of repair proteins to damaged DNA.

### Morphology and inner compartmentalization of DNA repair foci

The spatial distribution of DNA repair foci or inner compartmentalization of repair proteins in these foci can be analyzed on an individual cellular level via conventional confocal microscopy or more precisely by superresolution techniques, such as STED (stimulated emission depletion microscopy). As previously discussed, DNA repair foci are formed by accumulated proteins, including γH2AX, 53BP1, MDC1 or BRCA1 [20,67]. Interestingly, these proteins are characterized by a specific arrangement in not only repair foci but also in the whole cell nucleus. For example, Chapman et al. [67] showed that focal 53BP1-positive IRIF occurs in G0/G1 cells; however, in the S phase of the cell cycle, the 53BP1-positive IRIF contains a high BRCA1 positivity in the inner part. This process leads to 53BP1 exclusion to the periphery of these repair foci. Therefore, the level of 53BP1 at DNA damage sites is reduced in the S phase. We confirmed a similar phenomenon when we investigated γH2AX-positive tiny foci that colocalize with 53BP1 and surround the BRCA1 protein which was focally accumulated in the cores of DNA damage foci (Figure 4A-C). These observations document that these foci are characterized by specific inner compartmentalization of repair proteins. Additionally, Reindl et al. [68] showed that Rad51 does not form a nanostructure, but Rad51-positive and highly compact foci are decorated by the 53BP1 protein. Interestingly, Chk1 and Chk2 effector kinases that accumulate at DNA damage sites rapidly dissociate and are diffusely dispersed throughout the cell nucleus. Moreover, Chk2 was found to be highly mobile in the cell nucleus irrespective of DNA damage, but the phosphorylation of Chk2 by ATM was only restricted to DNA lesions [69,70]. Based on these data, it is evident that some repair proteins are immediately recruited to DNA lesions and then rapidly dissociate from these regions or relocalize on the periphery of DNA repair foci. These nuclear events may also be cell type- and cell cycle-specific or differences in the inner compartmentalization of repair foci could be caused by distinct doses and types of radiation. Alternatively, the spatial distribution of DNA repair proteins or their recruitment kinetics may be affected by genome instability, as shown by [20].

Studying the nuclear architecture, we have also documented that 53BP1 positivity is highly pronounced in so-called interchromatin granule-associated zones (IGAZs) and that 53BP1-positive spontaneous DNA lesions are located in close proximity to SC35-positive nuclear speckles [71]. These nuclear regions, which are well visible using electron microscopy, are considered the major nuclear bodies responsible for the storage and recycling of splicing factors [72,73]. The recruitment of DNA repair proteins to nuclear speckles has also been reported by Campalans et al. [74] in cells exposed to oxidative stress. This observation implies that nuclear speckles are, to some extent, involved in DNA repair processes and it seems likely that nuclear speckles may serve as reservoirs of some DNA repair proteins.

Yamauchi et al. [75] investigated another positional aspect of DNA repair foci. They showed clustering of focally arranged DNA lesions, which increased when Ku80, DNA-dependent protein kinases (DNA-PKcs), and ATM kinase were absent. In contrast, the depletion of 53BP1 reduces the number of nuclear foci consisting of DSBs. Interestingly, these foci were paired more frequently in heterochromatin regions than in euchromatin-rich nuclear domains. This finding indicates that the degree of chromatin condensation may affect the formation of DNA repair foci. Moreover, Falk et al. [76] showed that γH2AX-positive foci protrude from the interior of the heterochromatin compartment to the heterochromatin periphery, which is characterized by a lower chromatin density. In this case, the 53BP1 protein penetrates into the interior of heterochromatic domains that undergo subsequent decondensation following cell exposure to radiation. Goodarzi and Jeggo [77] reported that the chromatin composition around DSBs significantly affects the efficiency of DNA repair. Therefore, the type of chromatin in close proximity to DSBs might substantially contribute to the efficiency of DNA repair. Goodarzi and Jeggo [77] further claimed that heterochromatin-linked nuclear superstructures restrict signaling that is involved in the DNA damage response. These data show that heterochromatic DSBs are rapidly rearranged and relocated to the boundary between heterochromatin and euchromatin regions. In this review article, Figs. 5A and B illustrate the localization of the 53BP1 protein in close proximity to clusters of centromeric heterochromatin (chromocenters) in irradiated cells or cells treated with DNA-damaging agents. Jakob et al. [78] also identified DSBs located around highly compacted regions of chromatin, which supports the claim of [79] showing the relocation of DSBs from the interior to the periphery of heterochromatic clusters. These authors documented that GFP-tagged XRCC1 (a marker of single-strand breaks in DNA) is recruited to chromocenters, likely surrounded by a mixture of distinct DNA lesions. During this DNA damage response inside heterochromatin clusters, histone H2AX was phosphorylated relatively early, and several minutes after DNA injury, γH2AX subsequently relocated to the periphery of chromocenters. Again, these results demonstrate the mobility of DNA lesions. Moreover, the localized kinetics of these lesions are presumably regulated by a mechanism dependent on ATM kinase. This molecular mechanism was documented by [77], showing that ATM signaling is responsible for the relaxation of heterochromatin in the vicinity of DSB sites. This process is required primarily for the repair of DSBs in heterochromatin that creates a “niche” essential for the binding of repair proteins to these genomic regions [77].

The morphology of DNA repair foci is distinct in different cell types and particularly after cell treatment with distinct genotoxic agents ([80]; also refer to Figure 5Ba-c). For example, camptothecin-treated cells (CPT) are characterized by tiny 53BP1-positive foci that are less robust than IRIF (Figure 5Bb, c [81];). Moreover, spontaneous DNA lesions (1-3 foci per cell nucleus) are strikingly larger than CTP-induced foci, and interestingly, the 53BP1 diffusion kinetics in these spontaneous lesions are different from those observed for IRIF or UV-induced DNA lesions (Figure 5a, b [53,60];). The effect of cytotoxic drugs and radiation can also be studied by following the number of DNA damage foci. For example, the morphology and number of DNA repair foci were investigated by [82]. These authors showed that cells of longer-lived species exhibit a higher number of 53BP1-positive foci than cells of shorter-lived species. An increase in the number of 53BP1 foci may be associated with reduced DNA fragmentation and a lower number of cells with micronuclei formation. These findings imply that longer-lived species are characterized by strengthened defense mechanisms against DNA injury and support the claim that there is a functional link between the processes of aging and DNA repair [83]. Moreover, Markova et al. [84] showed that the number of endogenous 53BP1 foci can be used as a marker of tumor cell radiosensitivity. These data fit well with our observation that tumors with a distinct mutation in TP53 genes show a distinct sensitivity to irradiation, which was manifested as distinct localized kinetics of 53BP1 at DNA lesions [20].

### Effects of histone deacetylases (HDACs) and inhibitors of HDACs or PARP on DNA repair processes

**Figure 6:**
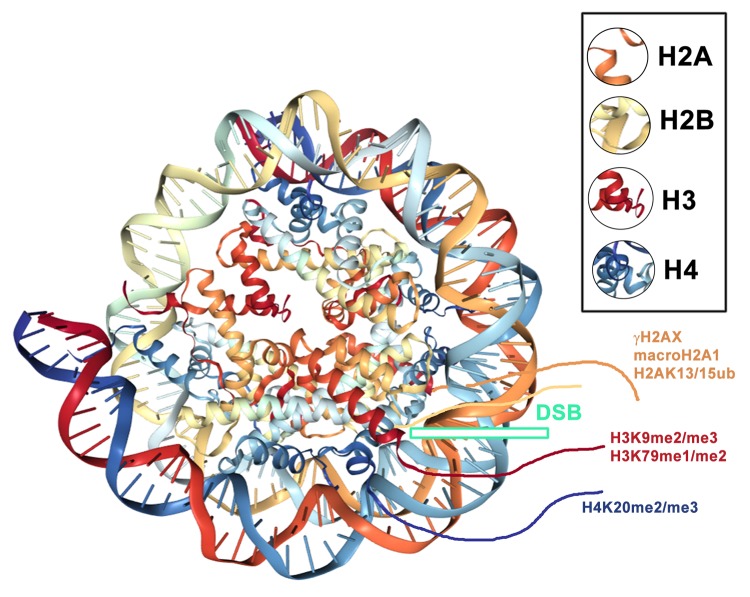
An example of a nucleosome with DSBs (green frame) and common histone posttranslational modifications that appear in close vicinity to DSB sites. Illustration of the nucleosome was adapted according to the PDB database; see http://www.rcsb.org/3d-view/3C1B/1.

Cann and Dellaire [85] noted that highly condensed heterochromatin is dedicated to protecting the genome against injury. However, chromatin compaction may represent an obstacle for proteins that must recognize damaged sites in DNA. Thus, chromatin in the vicinity of DNA lesions should be highly relaxed. Burgess et al. [86] showed a rapid but transient expansion of irradiated chromatin, which is an essential step for the activation of physiological DNA repair pathways. In this regard, the functioning of histone acetyltransferases (HATs) and, mainly, the clinical applications of inhibitors of histone deacetylases (HDACi) may contribute to the DNA damage response, accompanied by chromatin decondensation. For example, the inhibition of HDACs enhances chromatin relaxation, which could increase DNA repair effectiveness when implemented around DNA lesions. Paradoxically, several proteins that recognize highly compact heterochromatin, including the Polycomb group (PcG)-related proteins BMI1 and Mel18 and heterochromatin protein 1 (HP1), are known to be recruited to DNA lesions [87–89]. These proteins likely play a role in the later stages of DDR when chromatin compaction appears [86]. However, we showed that the BMI1 protein accumulates at DNA lesions immediately after local laser microirradiation [90]. Importantly, the shift from decondensed to compact chromatin requires the ATM-dependent accumulation of macroH2A1 and the tumor suppressor PRDM2 at DNA lesions to promote DSB-flanking H3K9 dimethylation ([91]; Figure 6). We showed that cell treatment with an HDAC inhibitor prevents the accumulation of not only BMI1 but also the H3K9 binding partner HP1β to microirradiated chromatin [90]. Based on this observation, we conclude that the degree of chromatin compaction and heterochromatin-like proteins affect the effectiveness of DNA damage responses. Furthermore, Han et al. [92] described that orchestrated chromatin condensation is essential for chromosome protection from DNA damage. This multilevel process is specific for distinct types of genome injury, including DSBs and CPDs. Han et al. [92] showed a slower repair of CPDs in heterochromatin compared with euchromatic regions. These results confirm the highly complicated and complex kinetics of factors involved in DNA repair machinery. This claim is also supported by a recently published paper indicating the repair of DSB sites via the 53BP1-dependent pathway. This study showed that the 53BP1-regulated repair mechanism predominantly appears in the heterochromatin compartment that is characterized by the presence of specific epigenetic markers, including transcriptionally repressive H3K9 trimethylation and H4K20 methylation ([93]; Figure 6). DDR-related functions of epigenetic factors, including chromatin modifiers, chromatin remodelers, histone markers, and histone chaperones, have also been described in a prime-repair-restore model published by [94]. This model includes three steps: (1) the access “prime” step for chromatin regulators; (2) the repair step mediated by DDR components; and (3) the restoration step mediated by new histone deposits and histone variant exchange. Such observations cumulatively show that DNA repair processes consist of hierarchical events that are affected by the degree of chromatin compaction [95].

An interesting example of chromatin decondensation can be found in embryonic stem cells (ESCs). In general, it is accepted that these cells are characterized by a more open chromatin configuration, which may be experimentally changed during ESC differentiation characterized by the opposite effect, chromatin compaction [96,97]. Venkatesh et al. [97] showed that the same dose of radiation causes more 53BP1-positive IRIF in human ESCs than in normal human fibroblasts. Moreover, in ESCs, the pluripotency transcription factor Oct4 seems to be an important player in DNA repair processes because we recently observed that the Oct4 protein is recruited to locally induced DNA lesions in mouse ESCs and that this recruitment is accompanied by H3K9 deacetylation. Moreover, ES cells were characterized by the recruitment of HDAC 1 to locally microirradiated chromatin [55]. In general, HDAC 1 and HDAC 2, which also deacetylate H4K16 at DSB sites, contribute to the regulation of the interaction between the 53BP1 protein and methylated histones [98,99]. Tang et al. [100] confirmed that H4K16 acetylation affects the binding of the 53BP1 protein to H4K20 dimethylated chromatin containing DNA lesions. Consistent with this observation, Miller et al. [99] showed that HDAC 1 and HDAC 2 are responsible for H3K56 deacetylation, and these enzymes are recruited to DSB sites, whereas inhibition of HDAC 1 and HDAC 2 reduces the accumulation of the 53BP1 protein at DNA lesions (summarized by [101]).

Interestingly, the depletion of 53BP1 alleviates the hypersensitivity of BRCA1 mutant cells to poly-ADP-ribose polymerase-1 inhibitors (PARPi) and restores repair by HDR [102]. In cells characterized by the depletion of both the BRCA1 and 53BP1 proteins, genomic stability is restored as a result of the recovery of a functional HDR pathway. Given this functionality, the 53BP1 level could also be used as a diagnostic tool in BRCA1-mutant tumors intended to be treated by PARP inhibitors, which represent promising anti-cancer drugs [27]. Moreover, the clinical use of PARP inhibitors could be reconsidered in tissues treated with radiotherapy. This claim is justified given that PARP inhibition prevents the recruitment of some proteins, including BMI1 and Mel18, to DNA lesions [88].

### Specific histone ubiquitination and methylation linked to the 53BP1 protein function at DNA lesions

Histone posttranslational modifications regulate the recruitment of 53BP1 to DNA lesions. In general, the specific histone signature is essential in the DNA damage response, where not only histone phosphorylation but also methylation and ubiquitination play important roles. Current models show that the accumulation of the 53BP1 protein at IRIF is dependent on (1) H2AK13/15-anchored ubiquitin chains generated by the E3 ubiquitin ligases RNF8 and RNF168 ([103]; Figure 2A, E and Figure 6) or (2) the direct interaction of the 53BP1 protein with dimethylated H4K20 ([2,104,105]; Figure 2A, D and Figure 3). H4K20me2 surrounds 53BP1-positive repair foci that also colocalize with accumulated MDC1 protein [2]. Suchánková et al. [20] also showed that robust MDC1-positive IRIF contain accumulated γH2AX in the interior of these foci that are also positive for BRCA1 ([20], and see example in Figure 4B). These data document that DNA repair proteins, including 53BP1, BRCA1, and histone posttranslational modifications, are specifically arranged in repair foci ([2,20]; Figure 4A-C). In Figure 4B, C, we illustrate that γH2AX is more dispersed inside and in close proximity to spontaneous DNA lesions, and the BRCA1 protein is particularly characterized by more focal accumulation. We also show that H4K20me2/me3 are dispersed within accumulated and robust 53BP1-positive DNA lesions, and these histone posttranslational modifications also appear in close proximity to DSB sites [4]. On the other hand, we have to take into consideration that the nuclear distribution pattern of DNA repair proteins, accumulated at repair foci, can also be affected by the type and dose of radiation.

DNA lesions are characterized by additional epigenetic features, for example, by H3K79 methylation [106]. For example, 53BP1 has been shown to be recruited to H3K79‑methylated regions. The first evidence of a mutual link between 53BP1 and H3K79 methylation was documented by [107], who showed that the tandem Tudor domain of the human 53BP1 protein recognizes methylated H3K79. Interestingly, the H3K79 methylation level is not changed in damaged genomic regions, likely as a result of chromatin relaxation at DSB sites. However, this epigenetic landscape enables the binding of the 53BP1 protein to damaged chromatin [107]. Additionally, H3K79 di-methylation is indispensable for 53BP1 recruitment because the main histone target, recognized by the 53BP1 protein at DSB sites, was determined to be H4K20 dimethylated [2,108–110]. Therefore, the most important histone posttranslational modification, decisive for repair functions mediated via 53BP1, is H4K20 methylation. However, methylation of H3K79 may be essential for 53BP1 recruitment to damaged chromatin when the H4K20me2 level is reduced, which may be the case for cells in pathophysiological states [109].

It is evident that 53BP1-positive foci colocalize or are surrounded by H4K20me2-dense chromatin [2,107,111]. However, H4K20 methylation is attractive for the 53BP1 protein only at DNA lesions and not when the gene expression is regulated via the 53BP1-p53 signaling pathway. In general, the process that leads to H4K20me2/me3 is mediated via Suv4-20h histone methyltransferase [112]. This fundamental epigenetic phenomenon was affirmed in Suv4-20h-double-null (dn) mice with perinatally lethal conditions as a result of the loss of H4K20me2 and H4K20me3. Interestingly, this nuclear event was accompanied by a genome-wide transition of H4K20me2/me3 to H4K20me1, which was regulated by PR-Set7 methyltransferase. This epigenetic change caused a higher cell sensitivity to genotoxic stress [112,113]. In HeLa cells, Pei et al. [114] documented a pronounced H4K20me2 at DNA lesions, which was mediated by another histone methyltransferase, called Multiple Myeloma SET protein (MMSET). It is known that the phosphorylation of MMSET is dependent on ATM kinase, and when this enzyme accumulates to DSB sites, *de novo* H4K20 di-methylation appears in damaged part of the genome. Additional experiments showed that the depletion of MMSET reduces H4K20 methylation at DNA lesions. Surprisingly, the recruitment of MMSET to DSB sites was dependent on the γH2AX-MDC1-mediated repair pathway. Therefore, a functional role of not only Suv4-20h HMTs but also MMSET must be considered in experiments revealing the functional properties of H4K20me2-dependent DNA repair. Chitale and Richly [115] also confirmed that MMSET mediates H4K20me2 at damaged chromatin. In these studies, the repair process was associated with the recruitment of the XPA factor to DNA lesions, consisting of NER-recognized CPDs. Therefore, H4K20me2 could likely play a role not only during 53BP1-mediated NHEJ repair of DSBs but also, to some extent, during other repair mechanisms, including NER.

Here, we additionally explain the results of [4], showing that H4K20me3 also plays a role in the DNA damage response. Surprisingly, this is H4K20me3, but not H4K20me1/me2, whose level is pronouncedly increased in locally microirradiated chromatin [4]. Furthermore, Li et al. [116] showed that depletion of PR-Set7, accompanied by loss of H4K20me1, results in the accumulation of DNA damage and cell cycle arrest, dependent on ATR function [116]. Based on these observations, it seems to be evident that H4K20 methylation is the potential target for the epi-drugs affecting not only epigenomic but also DNA repair processes [117,118].

The repair of DSBs also requires the methylation of histone H3 at the lysine 9 position (H3K9me3), as shown by [119]. However, the mechanism by which H3K9me2 and H3K9me3, as HP1-binding partners, regulate the DNA damage response remains unknown. Burgess et al. [86] revealed that Suv39h HMTs recruit 53BP1-positive DNA lesions to tri-methylate H3K9. Furthermore, Khurana et al. [91] reported that laser irradiation induced the accumulation of the tumor suppressor PRDM2 and increased the level of H3K9 dimethylation at irradiated chromatin. These authors identified PRDM2 and macroH2A1 (Figure 6) as ATM-dependent components of DSB repair mediated via the HDR pathway. They showed that the loss of macroH2A1 or PRDM2 or chromatin decondensation affected the retention of the BRCA1 protein but not 53BP1 at DSB sites. Moreover, the macroH2A1/PRDM2 complex regulates chromatin condensation, and the function of these proteins is linked to H3K9 dimethylation, which is associated with DSBs. Additionally, Ayrapetov et al. [119] reported that H3K9 methylation in close proximity to DNA lesions appears due to the DNA repair function of Suv39h1 methyltransferase. This process involves dynamic changes in H3K9 methylation in euchromatin and is essential for the remodeling of damaged genomic regions. Chen and Zhu [120] also summarized that pronounced H3K9me3 levels occur near DSBs that appear in euchromatin. Luijsterburg et al. [89] indicated that H3K9me3 is not necessary for the recruitment of the heterochromatin protein HP1β to DNA lesions. Interestingly, a complex that contains Kap-1, HP1 protein, and the Suv39h1 methyltransferase relocate to the chromatin in the vicinity of DSBs. This process is responsible for H3K9 methylation in a PARP1-dependent manner. H3K9me3 also initiates the activation of Tip60 acetyltransferase, which acetylates both ATM kinase and histone H4. This epigenetic event leads to the induction of the open chromatin configuration that appears specifically at UV-induced DNA lesions [95].

We have recently identified H3K9 deacetylation and the recruitment of histone deacetylase 1 (HDAC 1) to UVA-microirradiated chromatin [55]. It is generally accepted that both histone deacetylases and sirtuins (SIRTs) participate in DNA repair processes. For example, Paredes and Chua [121] have shown that SIRT7 is recruited by PARP1 to DSB sites, which leads to changes in H3K18 acetylation at damaged chromatin. This epigenetic event enables the accumulation of the 53BP1 protein at H3K18 deacetylated chromatin within DSBs, recognized by proteins from the NHEJ repair pathway [121,122].

Locally induced DNA lesions are also positively identified by H3K27me3, a marker of heterochromatin. This histone posttranslational modification represents a binding partner for the BMI1 and Mel18 proteins. These proteins also accumulate at UV-induced DNA lesions [88,90]. Enhancer of Zeste protein-2 (EZH2), a component of Polycomb Repressive Complex 2 (PRC2), catalyzes H3K27me3. O'Hagan et al. [123] showed that oxidative damage increases the interaction of EZH2 with DNA methyltransferase 1 (DNMT1). Additionally, Campbell et al. [124] documented that PRC2 was recruited to DNA damage sites, while this was not linked to the phosphorylation of H2AX at these chromatin regions. The recruitment kinetics of PRC2 is dependent on PARP activity, and the depletion of EZH2 weakened the repair of DSBs and increased the cell sensitivity to γ-rays [124].

Several studies have also indicated a function of the ubiquitinylation of the histones H2A, H2B, and H2AX in DNA damage response. This process is mediated via the ubiquitin E3 ligase RNF8, which is responsible for the focal accumulation of various DNA repair-related factors in IRIF, consisting of 53BP1, PTIP or BRCA1 proteins [125–133]. Interestingly, H2AX ubiquitinylation by proteins from the PRC1 complex contributes to 53BP1 and BRCA1 recruitment to chromatin with DSBs ([134]; Figure 6). Furthermore, the sumoylation of 53BP1 and BRCA1 by PIAS1 and PIAS4 SUMO E3 ligases enhances the residence times of 53BP1 and BRCA1 at DNA damage foci (summarized by [130]). Hu et al. [135] identified the ubiquitin recognition mechanisms in the nucleosome. They showed the regulation of 53BP1 via pro-activation functions of the ubiquitin ligase RNF168 and the inhibitory function of the ubiquitin ligases RNF169 and RAD18. Wilson et al. [136] also documented the mechanism of DNA repair that is mediated by histone ubiquitination. Gatti et al. [137] and Mattiroli et al. [138] showed that RNF168 ubiquitinates histone H2A on lysine 13 and lysine 15. This epigenetic process affects the accumulation of the 53BP1 protein in chromatin with DSBs. Direct and selective binding of 53BP1 to ubiquitinated H2AK15 occurs via the ubiquitination-dependent recruitment motif of 53BP1 ([139]; Figure 2A, E). This process works in parallel with H4K20me2, which requires the functional Tudor domain of 53BP1 (Figure 2A, C, D). An important role is also ascribed to the Tudor-interacting repair regulator (TIRR), which directly binds to the tandem Tudor domain of 53BP1 and mimics its H4K20me2-binding properties [140]. When ATM phosphorylates 53BP1 and recruits RIF1, the complex consisting of 53BP1-TIRR is abrogated. Moreover, overexpression of TIRR weakens the function of 53BP1, and TIRR depletion destabilizes the 53BP1 protein when dissolved in the nucleoplasm. These experiments show that TIRR significantly regulates the function of 53BP1 [140]. Wang et al. [141] observed that the loop of the TIRR protein interacts with the 53BP1 tandem Tudor domain and thus mimics the methylated lysine-binding region in this domain. Thus, TIRR seems to also be the main competitor of H4K20 methylation when attracting the 53BP1 protein. Dai et al. [142] precisely identified a mechanism by which TIRR recognizes 53BP1 foci.

### 53BP1 functions in *Igh* class switch recombination

It is well known that the 53BP1 protein is also a factor that plays a role in *Igh* class switch recombination (CSR) in B lymphocytes and is an essential target for sensitizing BRCA1-deficient tumors to PARP inhibitors [143]. 53BP1 contributes to DNA repair and the orientation of the broken DNA ends during class-switch recombination [144,145]. It was reported that after depletion of the 53BP1 protein, the function of CSR is significantly abrogated [146,147].

It is well known that *Igh* class switch recombination (CSR) replaces one set of *Igh* constant region exons (CHs) with another. Using this mechanism, mature B lymphocytes can change the class of expressed antibodies from IgM to IgG, IgA, or IgE through a recombination/deletion process. CSR is induced by activation-induced cytidine deaminase, which initiates a cascade of nuclear processes that lead to DNA double-strand break formation in switch regions. In mature B cells, 53BP1-dependent CSR occurs via an intrachromosomal looping and deletion mechanism [148,149]. This process works in parallel with a specific histone signature, and H4K20 methylation is a very important key player that is specific for DNA repair in the immune system [150].

### The DNA repair-related function of the 53BP1 protein is associated with the function of lamins

A-type lamins are important components of nuclear architecture. Redwood et al. [151] showed that the depletion of A-type lamins is involved in the degradation of the 53BP1 protein in DNA repair. Gonzalez-Suarez et al. [152] also demonstrated that A-type lamin-deficient cells are characterized by a lower level of 53BP1 in comparison to their wild-type counterpart. Noda et al. [83] documented that the lamin A- or progerin-associated nuclear envelope takes part not only in cellular aging but also in DNA repair processes. In Hutchinson-Gilford progeria syndrome (HGPS) cells, which are characterized by a mutation in the *LMNA* gene encoding A-type lamins, residual unrepaired DSBs appear. DNA lesions in laminopathy cells are recognized by defecting long-range NHEJ. This error-prone process may lead to an abrogated function of telomeres. A functional role of the 53BP1 protein in telomere maintenance was described by [153], who showed that depletion of the shelterin protein TRF2 activates ATM kinase, and the 53BP1 protein is recruited to unprotected chromosome ends that are recognized as DSBs. These unprotected telomeres are highly mobile, and their rearrangement requires both ATM and the 53BP1 protein. However, a fully functional NHEJ repair mechanism is not activated.

**Figure 7:**
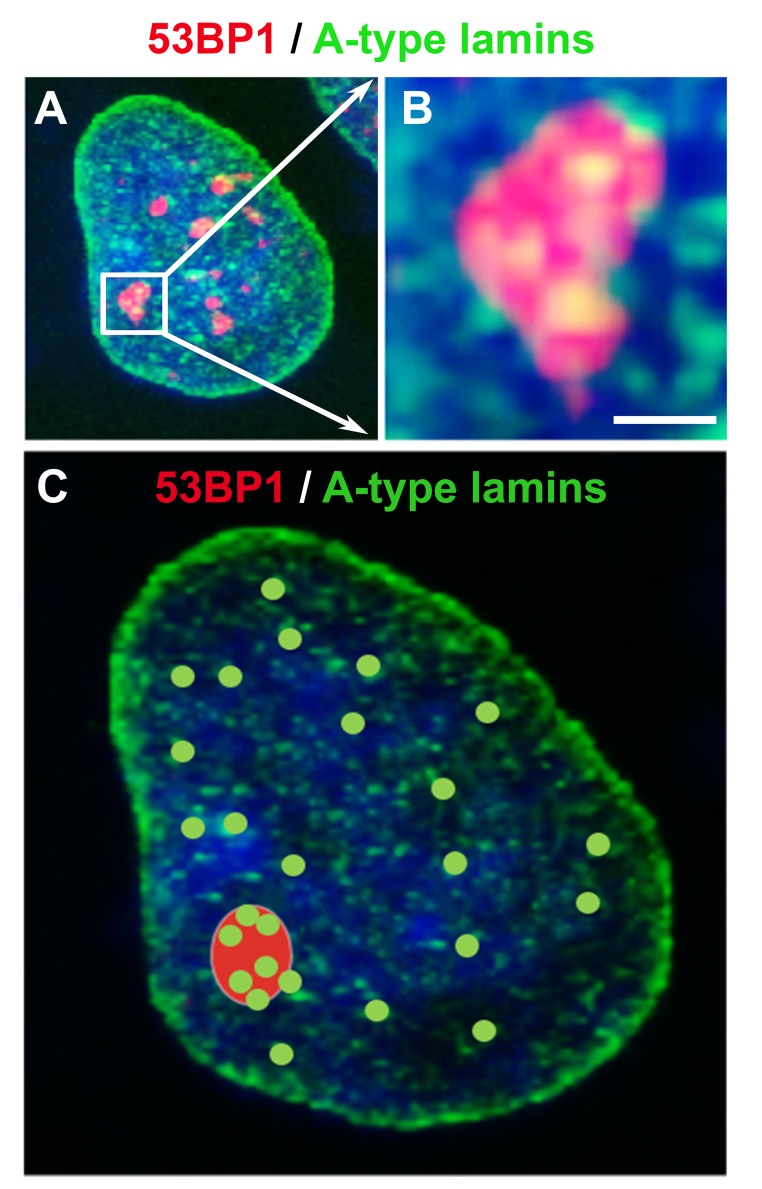
The nuclear distribution pattern of A-type lamins (green) in 53BP1-positive DNA repair foci (red). (**A**) Spontaneously occurring DNA lesions in HeLa cells are shown, and (**B**) magnification is delineated by white arrows. Although A-type lamins do not directly accumulate at DNA lesions, (**C**) lamin A/C positivity in spontaneous DNA lesions could be essential for the stability of these 53BP1-positive foci and their error-free repair (refer to primary data and methodology in [155,156]). Scale bars, 0.5 µm.

A-type lamins also affect the efficiency of the short-range repair of DSBs induced by ionizing radiation. Redwood et al. [154] suggested that A-type lamins may be components of the HDR mechanism and that lamin-deficient cells are characterized by increased radiosensitivity. Gibbs-Seymour et al. [155] reported that 53BP1 is a lamin A/C-binding protein and that the interaction between 53BP1 and A-type lamins is mediated via the Tudor domain of the 53BP1 protein. A link between these proteins is also supported by the finding that the physiological levels of lamins A/C are necessary for the physiological level of the 53BP1 protein. Moreover, lamins in the nuclear interior facilitate the recruitment of 53BP1 to DNA lesions although lamins do not accumulate at locally irradiated chromatin [155,156]. However, we showed that 53BP1-positive foci colocalize or are decorated by homogeneously distributed internal lamin A/C deposits ([156]; for illustration see Figure 7A-C). Therefore, it is possible that internal lamins A/C anchor and stabilize 53BP1-positive DNA damage foci.

The dynamics and regulatory function of lamins A/C after DNA damage have also been characterized by [157], who showed that lamin A interacts with chromatin via the phosphorylated form of H2AX. Moreover, the depletion of A-type lamins reduces the stability of DNA repair foci [157] and decreases the accumulation of the 53BP1 protein at UVA-induced DNA lesions [158]. These data indicate that intact lamins are important for the maintenance of the architecture of DNA repair foci. Interestingly, the abrogation of lamin function causes chromatin decondensation and rearrangement of the 53BP1 protein at DNA lesions. Gibbs-Seymour et al. [155] also documented that lamins A/C interact with the 53BP1 protein under normal physiological conditions; however, DNA damage weakens this protein-protein interaction. In this case, the 53BP1 protein is degraded in the 26S proteasome, but the depletion of the ubiquitin-conjugating enzyme UbcH7 restores the lamin A/C-53BP1 complex [159]. Interestingly, the lamin precursor pre-lamin A interferes with damaged chromatin [160]. It has been shown that the pre-lamin A level increases following DNA damage, and lamins A/C or PML bodies serve as a scaffold that regulates the nuclear organization of DNA repair foci [160]. Additionally, we recently showed that PML deficiency affects the local motion of 53BP1-positive repair foci and alters the composition and number of IRIF [71].

Noda et al. [83] addressed DDR-related processes in laminopathy cells focusing on telomerase function. These authors introduced the *TERT* gene into HGPS cells, which led to cell immortalization. Interestingly, the irregular shape of HGPS cells was changed to a shape that is characteristic of cells with a normal physiological function. In this case, the number of 53BP1 repair foci was reduced. Noda et al. [83] summarized that the observed effect was a consequence of DSBs that could not be repaired in nondividing cells. It is possible that this process is regulated via telomerase expression. Another possibility is that telomerase might prevent the formation of spontaneous DNA lesions in HGPS cells [83]. The authors also showed that heterochromatic regions in the vicinity of the nuclear membrane of laminopathy cells consist of newly formed DSBs or unrepaired DNA lesions. This nuclear event may initiate a reorganization of the nuclear architecture that is characterized by the formation of nuclear blebs in A-type lamin-deficient cells. These results unambiguously show that A-type lamins not only guard physiological cell aging but also regulate chromatin compaction around DNA lesions. The processes of physiological cell aging and physiological DNA repair seem to be mutually connected, particularly via the function of A-type lamins and their associated proteins.

## CONCLUSIONS

53BP1 is an important protein of double-strand break repair because of its interaction with damaged chromatin, characterized by specific epigenetic markers, including H2AK15 ubiquitination, phosphorylation of H2AX, methylation of H3K9 and H3K79 or H4K20 di-/tri-methylation [114]. The abovementioned epigenetic features are essential for physiological DNA damage repair. In the case of pathophysiological processes accompanied by error-prone DNA repair mechanisms, epi-drugs, including inhibitors of HDACs or PARP, represent very promising therapeutic tools for adapting the epigenetic landscape to mediate successful DNA repair processes. The data summarized herein provide a short overview of the histone signature and dynamic protein compartmentalization inside and in close vicinity of DNA repair foci. We mainly focused on the structural and functional properties of the 53BP1 proteins and their interacting partners which play a role in the DNA damage response. Although 53BP1 is not the factor for the first “choice” of DNA repair, this protein is an essential key player of the NHEJ repair pathway, whose functional properties are significantly regulated via specific histone signatures.
